# Strategies to Improve Convolutional Neural Network Generalizability and Reference Standards for Glaucoma Detection From OCT Scans

**DOI:** 10.1167/tvst.10.4.16

**Published:** 2021-04-15

**Authors:** Kaveri A. Thakoor, Xinhui Li, Emmanouil Tsamis, Zane Z. Zemborain, Carlos Gustavo De Moraes, Paul Sajda, Donald C. Hood

**Affiliations:** 1 Department of Biomedical Engineering, Columbia University, New York, NY, USA; 2 Department of Psychology, Columbia University, New York, NY, USA; 3 Department of Ophthalmology, Columbia University, New York, NY, USA; 4 Department of Electrical Engineering, Columbia University, New York, NY, USA; 5 Department of Radiology (Physics), Columbia University, New York, NY, USA

**Keywords:** convolutional neural networks, glaucoma, optical coherence tomography, retinal nerve fiber layer, b-scan, reference standards

## Abstract

**Purpose:**

To develop and evaluate methods to improve the generalizability of convolutional neural networks (CNNs) trained to detect glaucoma from optical coherence tomography retinal nerve fiber layer probability maps, as well as optical coherence tomography circumpapillary disc (circle) b-scans, and to explore impact of reference standard (RS) on CNN accuracy.

**Methods:**

CNNs previously optimized for glaucoma detection from retinal nerve fiber layer probability maps, and newly developed CNNs adapted for glaucoma detection from optical coherence tomography b-scans, were evaluated on an unseen dataset (i.e., data collected at a different site). Multiple techniques were used to enhance CNN generalizability, including augmenting the training dataset, using multimodal input, and training with confidently rated images. Model performance was evaluated with different RS.

**Results:**

Training with data augmentation and training on confident images enhanced the accuracy of the CNNs for glaucoma detection on a new dataset by 5% to 9%. CNN performance was optimal when a similar RS was used to establish labels both for the training and the testing sets. However, interestingly, the CNNs described here were robust to variation in the RS.

**Conclusions:**

CNN generalizability can be improved with data augmentation, multiple input image modalities, and training on images with confident ratings. CNNs trained and tested with the same RS achieved best accuracy, suggesting that choosing a thorough and consistent RS for training and testing improves generalization to new datasets.

**Translational Relevance:**

Strategies for enhancing CNN generalizability and for choosing optimal RS should be standard practice for CNNs before their deployment for glaucoma detection.

## Introduction

Glaucoma is a leading cause of irreversible blindness worldwide, projected to affect 112 million people by 2040.^1^ If left untreated, glaucoma can ultimately lead to blindness. Although methods exist to diagnose and slow the progression of the disease, one of the greatest challenges is that more than one-half of cases remain undetected owing to a lack of timely assessment by a specialist.^2^ Furthermore, even among clinicians, there is no clear litmus test for a glaucoma diagnosis.^3^^,^^4^ Artificial intelligence has the potential to help expedite glaucoma detection and/or triage when access to specialist time may be limited. In addition, artificial intelligence may aid in prioritizing cases that need attention first, ensuring that care is given to those subtle or uncertain cases most requiring expert inspection. Although significant advances have been made in developing deep learning models for ophthalmology applications,^5^ there are two major issues that need to be addressed. First, how does one evaluate the generalizability of these models, and second, how does one choose reference standards (RS) for their validation?

A number of studies have developed approaches based upon convolutional neural networks (CNNs) to detect glaucoma from optical coherence tomography (OCT) images.^6^^–^^10^ These studies, in general, show excellent performance in terms of reasonably high sensitivity and specificity. However, to demonstrate clinical usefulness, it is essential to test a deep learning system's ability to be effective with a new dataset from a different clinic. Although deep learning approaches applied to fundus images have exhibited generalizability,^11^^,^^12^ most of the existing studies that focused on applying CNNs to OCT images lacked an evaluation of model performance on data collected from different OCT machines and/or at different locations. Our primary purpose here is to develop and evaluate methods to improve the generalizability of CNNs trained to detect glaucoma.

A second critical issue in determining the clinical usefulness of a deep learning model is the RS on which its accuracy is based.^5^ Because there is no litmus test for glaucoma detection, models are typically tested against an RS to determine their efficacy in detecting glaucomatous damage. However, there is no universal agreement on an RS; different studies have used different RS, sometimes using a different RS for training and testing. Our secondary purpose here is to explore the consequences of using different RS.

To address these issues, we build on prior work in which we developed CNNs that showed high accuracy (≥95%) for detecting glaucoma from OCT retinal nerve fiber layer (RNFL) probability map input.^6^^,^^7^ Here we develop models for glaucoma detection from OCT circumpapillary disc (circle) b-scan image input as well. Second, we evaluate the generalizability of the RNFL map model and b-scan models on a new dataset collected at a different location than the training dataset. Third, we describe and assess methods to improve the generalizability of both the RNFL map model and b-scan models. Finally, we measure the impact of choice of RS on CNN accuracy.

## Methods

### Datasets and RS for RNFL Map Models

#### RNFL Map Dataset (DS_RNFL-Map_)

**Figure 1.**
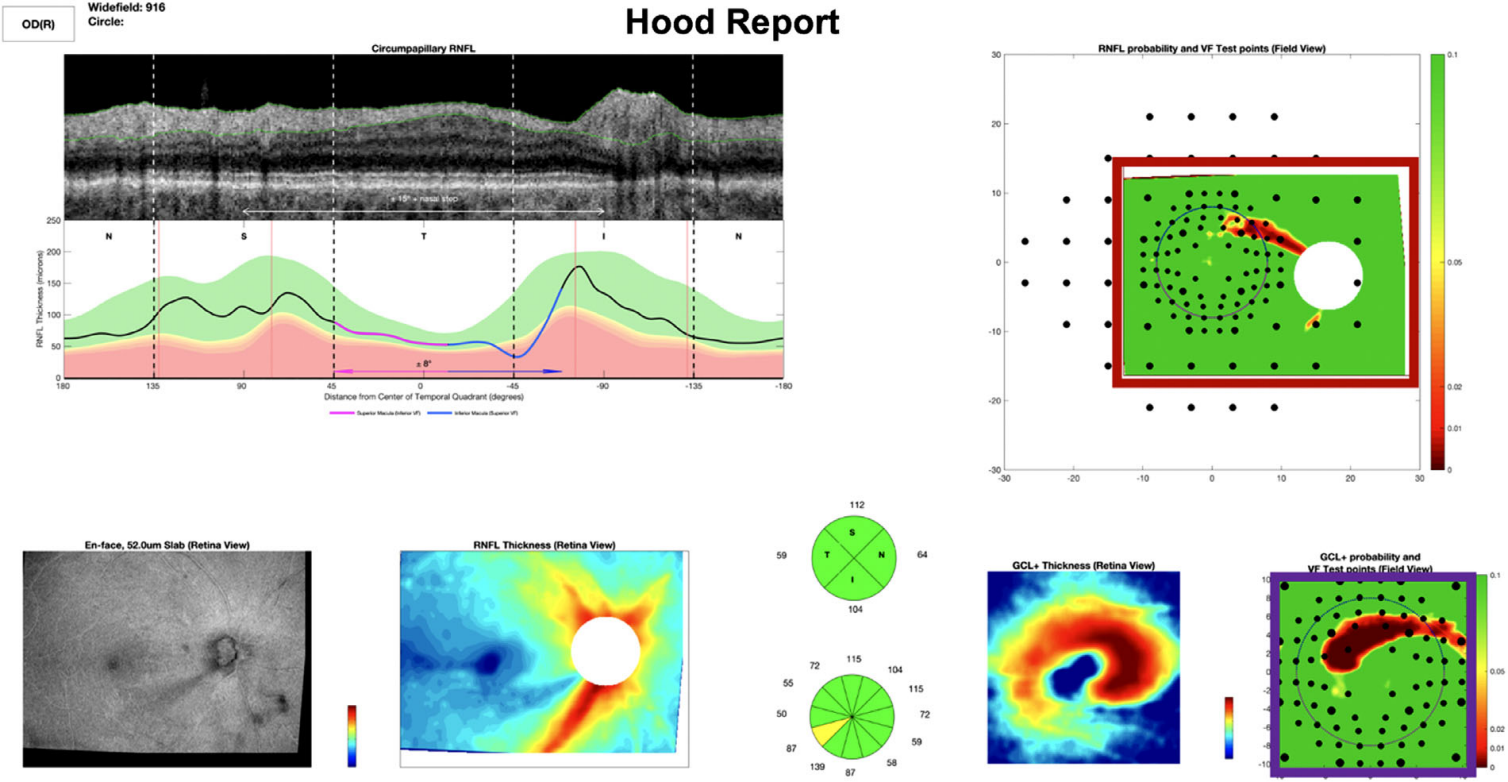
Example full Hood report which served as RS1_RNFL-Map_. A combination of the red and violet rectangles above served as RS2_RNFL-Map_. The red rectangle alone served as RS3_RNFL-Map_.

The OCT DS_RNFL-Map_, as described elsewhere,^6^ was composed of 737 eyes from wide-field Topcon Atlantis (Topcon, Inc, Tokyo, Japan) OCT cube scans collected in our laboratory as well as the machine's normative database (for healthy controls). Patients were early glaucoma or glaucoma suspects (mean deviation on 24-2 visual field better than –6 dB). Each widefield scan contained RNFL and retinal ganglion cell plus inner plexiform layer (RGCP) probability/deviation values over a 9 × 12 mm region, which included the fovea and optic disc. Figure 1 shows examples of the RNFL map (red rectangle) and the RGCP map (violet rectangle).

#### RNFL Map Generalizability Set (GS_RNFL-Map_)

The new dataset of RNFL maps used for generalizability testing, GS_RNFL-Map_, was collected on a Topcon Atlantis (Topcon, Inc, Tokyo, Japan) OCT machine at a different location (Columbia University Medical Center, Harkness Eye Institute) and by a different operator (than DS_RNFL-Map_), and was composed of RNFL probability maps from 135 healthy controls, glaucoma suspects, or patients with early glaucoma (24-2 mean deviation better than –6 dB; median, −1.67 dB; range, −5.62 to 0.84 dB; similar to that of DS_RNFL-Map_ with a median of −2.22; range, −4.69 to −0.49; the median patient age of 53 ± 16 years was also similar to that of DS_RNFL-Map_ of 57 ±13 years; further characteristics described in detail in prior work).^14^^,^^15^ See compositions of all datasets in the Supplementary Table S1a.

#### Reference Standards

We evaluated the CNN performance on GS_RNFL-Map_ based on four different RS. For each RS, the expert(s) gave a rating after reviewing the following information: RS1_RNFL-Map_, a custom commercial OCT report (Topcon,^13^ example in Fig. 1); RS2_RNFL-Map_, RNFL and RGCP probability maps (red and violet rectangles in Fig. 1); RS3_RNFL-Map_, RNFL probability maps alone (red rectangle only in Fig. 1); and RS4_RNFL-Map_, OCT as well as visual field information.

For RS1_RNFL-Map_ to RS3_RNFL-Map_, the ratings of a single OCT expert (DCH) were used, whereas RS4_RNFL-Map_ was based on a consensus of multiple experts. In each case, the expert(s) rated each patient eye on a scale between 0% and 100%, where “nonglaucomatous” was <50% and “glaucomatous” was >50%. For each of the 737 eyes in DS_RNFL-Map_, a single OCT expert (DCH) viewed a whole 3D Wide Glaucoma Report with VF [Visual Field] test points (Hood report), hereafter called a Hood report (equivalent to RS1_RNFL-Map_ as described elsewhere in this article), arriving at 544 nonglaucomatous and 193 glaucomatous RNFL maps, as previously described.^6^ The 135 eyes in GS_RNFL-Map_ were categorized as 78 nonglaucomatous and 57 glaucomatous based on RS4_RNFL-Map_ and as 81 nonglaucomatous and 54 glaucomatous based on RS1_RNFL-Map_.

### Datasets and RS for B-Scan Models

#### B-Scan DataSet (DS_B-Scan_)

**Figure 2.**
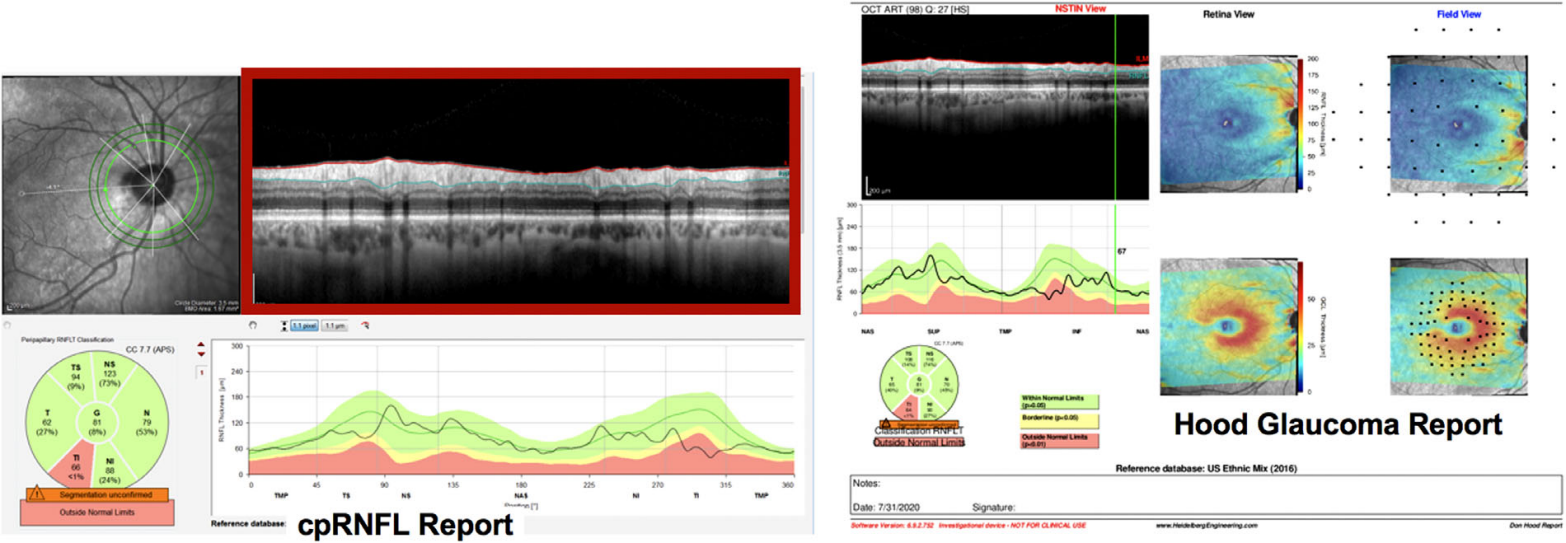
(A) Example cpRNFL report (left) and (B) full Hood Glaucoma Report (right). The cpRNFL report served as RS1_B-Scan_. The full report served as RS2_B-Scan_, and the b-scan alone (shown in red rectangle) served as RS3_B-Scan_.

The b-scan dataset (DS_B-Scan_) was composed of 3.5 mm circle b-scans from 771 scans (from 771 eyes) collected with a Heidelberg Spectralis OCT (Heidelberg Engineering, Inc., Heidelberg, Germany). We also generated the circumpapillary RNFL (cpRNFL) thickness profile for each scan. The orientation of these thickness profiles was in T (temporal)–S (superior)–N (nasal)–I (inferior)–T (temporal), following the same format as the commercial Heidelberg cpRNFL reports (Fig. 2A), whereas the commercial full Hood Glaucoma Report (Heidelberg Engineering, Inc.) in Figure 2B was in N (nasal)–S (superior)–T (temporal)–I (inferior)–N (nasal).

#### B-Scan Generalizability Set (GS_B-Scan_)

The new b-scan dataset used for generalizability testing, GS_B-Scan_, was collected on a different Heidelberg Spectralis OCT instrument at a different location (Columbia University Medical Center, Harkness Eye Institute) and by a different operator (than DS_B-Scan_), and was composed of 127 circle b-scans from 127 eyes (median mean deviation, −1.67 dB; range; −5.62 to 0.84dB, similar to that of DS_B-Scan_ with a median of −2.22; range, −4.69 to −0.49; median patient age of 53 ± 16 years, similar to that of DS_B-Scan_ of 57 ± 13 years).^15^ See the b-scan dataset composition in Supplementary Table S1b.

#### Reference Standards

Just as for RNFL maps, we evaluated the CNN performance on GS_B-Scan_ based on four different RS. For each RS, the expert(s) gave a rating after reviewing the following information: RS1_B-Scan_, cpRNFL reports only; RS2_B-Scan_, Heidelberg reports; RS3_B-Scan_, b-scans only; and RS4_B-Scan_, OCT as well as visual field information. Just as with RNFL maps, RS1_B-Scan_ to RS3_B-Scan_ were based on ratings of a single OCT expert (DCH), whereas the RS4_B-Scan_ was based on a consensus of multiple experts. In each case, the expert(s) rated each patient eye on a scale between 0% and 100%, where “nonglaucomatous” was <50% and “glaucomatous” was >50%. For each of the 771 eyes in DS_B-Scan_, a single OCT expert (DCH) viewed the commercial cpRNFL report (same as RS1_B-Scan_ described elsewhere in this article) and shown in Figure 2A, arriving at 474 nonglaucomatous and 297 glaucomatous b-scans. The 135 eyes in GS_B-Scan_ were categorized as 72 nonglaucomatous and 55 glaucomatous based on RS4_B-Scan_ and as 61 nonglaucomatous and 66 glaucomatous based on RS1_B-Scan_. For both RNFL maps and b-scans, although experts provided continuous ratings (between 0% and 100%), a final binary classification used for RS and for deep learning models was based on binary labels (glaucomatous >50% or nonglaucomatous <50%).

This study was approved by the Columbia University Institutional Review Board and adheres to the tenets set forth in the Declaration of Helsinki and the Health Insurance Portability and Accountability Act. Written informed consent was obtained from all subjects. The clinical trial associated with this study was registered at ClinicalTrials.gov (identifier: NCT02547740).

### Models

#### Models for RNFL Maps

Of the models described in previous work,^6^ the best-performing CNN (identified hereafter as ‘CNN A’) was ResNet18 + Random Forest, determined using repeated measures analysis of variance and Holm–Sidak corrected *t* tests.^16^ Performance of CNN A was tested on GS_RNFL-Map_ after (1) training on DS_RNFL-Map_ (as previously described)^6^ and after (2) improvement techniques described in the following sections.

#### Models for Circumpapillary B-scan Images

Building on the models described in previous work,^6^ we developed two new models (CNN B, trained on OCT data alone; and CNN C, pretrained on natural images).^17^ These models were evaluated for their performance on GS_B-Scan_ after (1) training on DS_B-Scan_ as described in the next section and after (2) improvement techniques described in this article.

CNN B and CNN C were independently trained, validated and tested on DS_B-Scan_ with a 60%:20%:20% ratio. To assess model generalizability, we tested the same models on GS_B-Scan_. CNN B was a lightweight model that was trained from scratch consisting of three convolutional blocks and two dense layers. Each convolutional block was composed of multiple two-dimensional convolutional filters, rectified linear unit or sigmoid activation, and a two-dimensional max pooling layer (see Supplementary Figures S1 and S2 in for more details, including strengths and weaknesses of the CNN B architecture). The hyperparameters of CNN B were fine tuned according to validation results. CNN C used ResNet50^18^ as the backbone, followed by a random Forest classifier. Models were built using the Python deep learning library, Keras (https://keras.io/), and were trained using Google's Colaboratory platform (https://colab.research.google.com/notebooks/) with GPU accelerator.

#### Techniques to Improve CNN Generalizability

**Figure 3.**
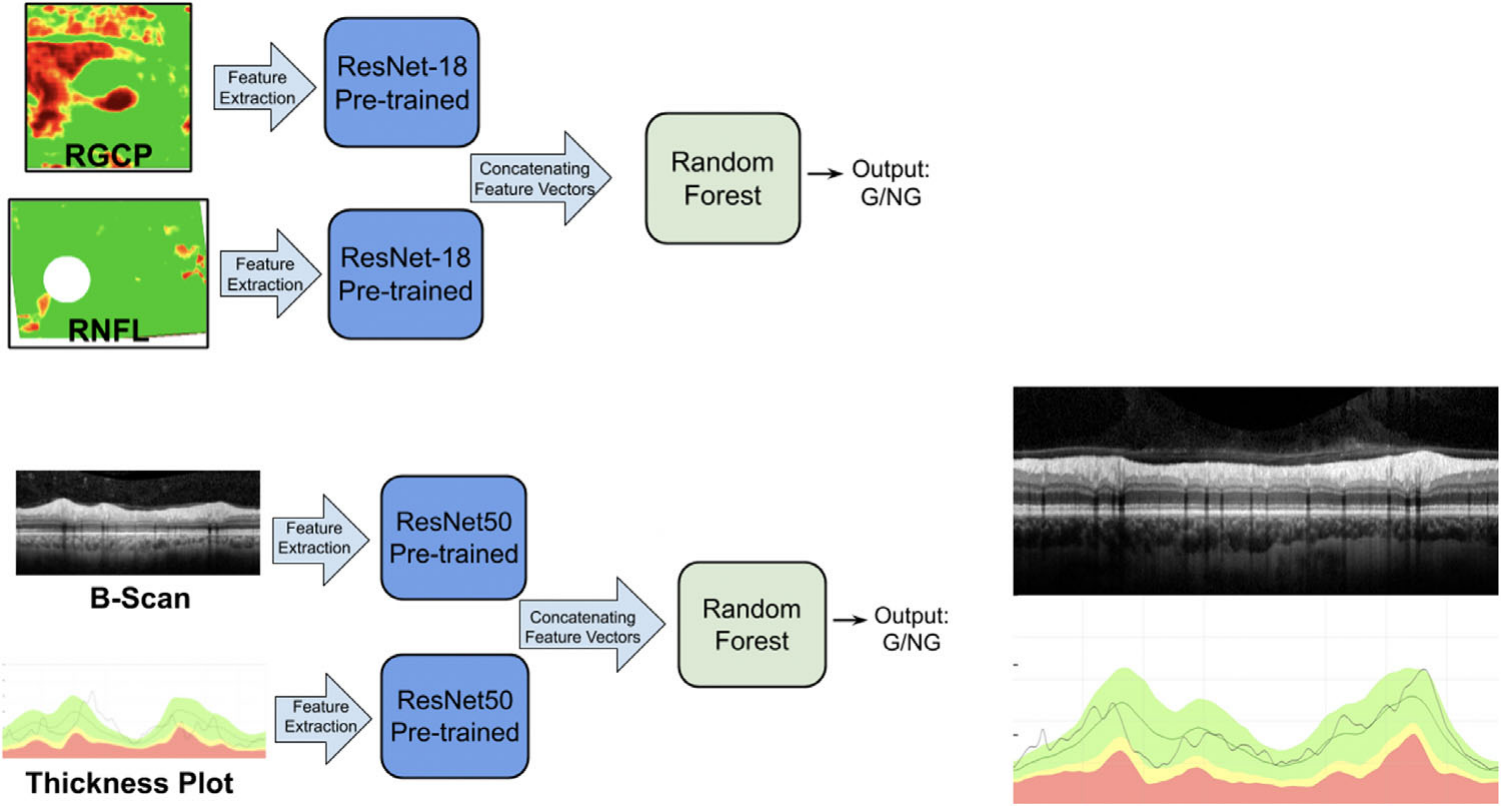
(A) Schematic showing the multimodal input image (feature concatenation) technique for RNFL + RGCP maps (top left) and for b-scans + thickness plots (bottom left). In each case, features were extracted from each image by a pretrained CNN and concatenated before being classified by a downstream Random Forest classifier. (B) At right is shown the image concatenation technique attempted for b-scans. The b-scans were vertically concatenated with thickness plots; these combined images were provided as input to the CNN.

We explored three techniques to improve generalizability. (1) Data augmentation involved increasing size of the training set by adding more images with size and scale variations consistent with machine differences. (2) For multimodal input images, we tried two multimodal techniques: (a) feature concatenation features were extracted from multimodal image types and then concatenated before being classified by a CNN. Specifically, for RNFL map images, CNN A was used to extract features from RNFL maps and RGCP maps, respectively. These features were then concatenated, and these combined features were classified as either glaucomatous or nonglaucomatous (as depicted in Fig. 3). For b-scan input, CNN C was used to extract features from b-scans and thickness plots, and the resulting concatenated features were classified as glaucomatous or nonglaucomatous (Fig. 3). (b) Image concatenation, for b-scan images, an additional multimodal image concatenation technique was attempted by placing b-scans vertically adjacent to thickness plots (similar in format to the clinical cpRNFL report shown in Fig. 2B). (3) Training on confidently rated (extreme) images: The model was trained on the images for which the expert gave confident ratings: a high glaucomatous probability rating (75%–100%) or a low glaucomatous probability rating (0%–25%).

#### Details: RNFL Map Improvement Techniques

Of the techniques described elsewhere in this article, data augmentation and multimodal input were used to improve generalizability for the RNFL map model, CNN A. Data augmentation was used to add training images with a 10% scale variation and with horizontal and vertical flips.^19^ This scaling variation was motivated by the fact that the instrument used to collect GS_RNFL-Map_ images had an 8% to 10% scaling difference from the machine used to collect DS_RNFL-Map_. Horizontal flips effectively added more left or right eyes, opposite of what existed in the training pool (because only one eye from each patient was present in DS_RNFL-Map_). Vertical flips also helped to augment training database size without loss of information or major modulation to existing RNFL maps. The second improvement technique consisted of multimodal image input: (a) just RNFL map features or (b) features concatenated from RNFL maps and RGCP maps (following the schematic shown in Fig. 3) were classified.

#### Details: B-Scan Improvement Techniques

Of the techniques described elsewhere in this article, data augmentation, multimodal input, and confident scans were used to improve generalizability for the best performing b-scan model, CNN C. In particular, the data augmentation variations included horizontal flips and vertical shifts, both plausible changes to account for collection of b-scan data on a different OCT machine. Both feature concatenation (Fig. 3A) and image concatenation (Fig. 3B) multimodal input techniques were attempted for b-scans. To evaluate how the confidence level of b-scan ratings impacted model performance, 703 circle b-scans which a single OCT expert (DCH) rated as glaucomatous with reasonable confidence (<25% = nonglaucomatous, n = 444; >75% = glaucomatous, n = 259) were selected from DS_B-Scan_ as a confident dataset (C-DS_B-Scan_). To evaluate potential improvement in generalizability, we reran the same process used previously (training–validation–testing on DS_B-Scan_, testing on GS_B-Scan_) with C-DS_B-Scan_ (detailed results shown in Supplementary Table S2).

**Figure 4.**
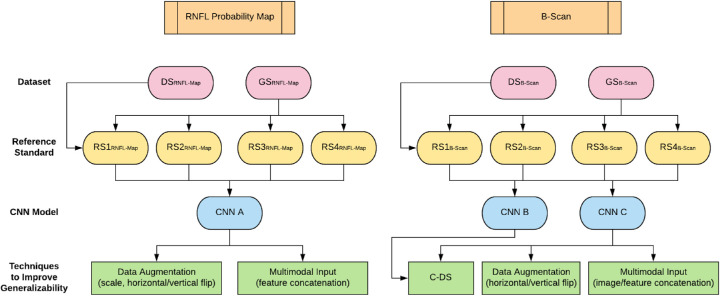
Flowchart showing terminology and methodology used in our study: OCT image types are shown in orange boxes at the top, followed by corresponding datasets (red ovals), RS (yellow ovals), models (blue ovals), and generalizability techniques (green rectangles) for RNFL map input (left) and b-scan input (right), respectively.

Figure 4 shows a flow chart of our full methodology for this paper: all generalizability improvement techniques and all RS used for each model type.

## Results

### Performance of the New B-Scan Models

The b-scan models, CNN B, trained on OCT data alone, and CNN C, pretrained on natural images, showed high and comparable accuracies, 94.4% (CNN B) and 95.8% (CNN C), for DS_B-Scan_ (Table 1, B-Scan Models, bottom half of table, Column 2), consistent with past studies.^6^^,^^20^ The performance of these b-scan models was also similar to the performance, 94.8%, of the RNFL map model, CNN A, using DS_RNFL-Map_. For both model types, we present generalizability set results using only RS1_RNFL-Map_/RS1_B-Scan_ and RS4_RNFL-Map_/RS4_B-Scan_, respectively, owing to the clinical relevance of these two RS. (Results for all models using RS2_RNFL-Map_/RS2_B-Scan_ and RS3_RNFL-Map_/RS3_B-Scan_ are presented in Supplementary Table S3).

**Table 1.** Accuracies of Best-Performing RNFL Map Model and New B-Scan Models on DS_RNFL-Map_ and DS_B-Scan_ as Well as on GS_RNFL-Map_ and GS_B-Scan_ With Varying RS

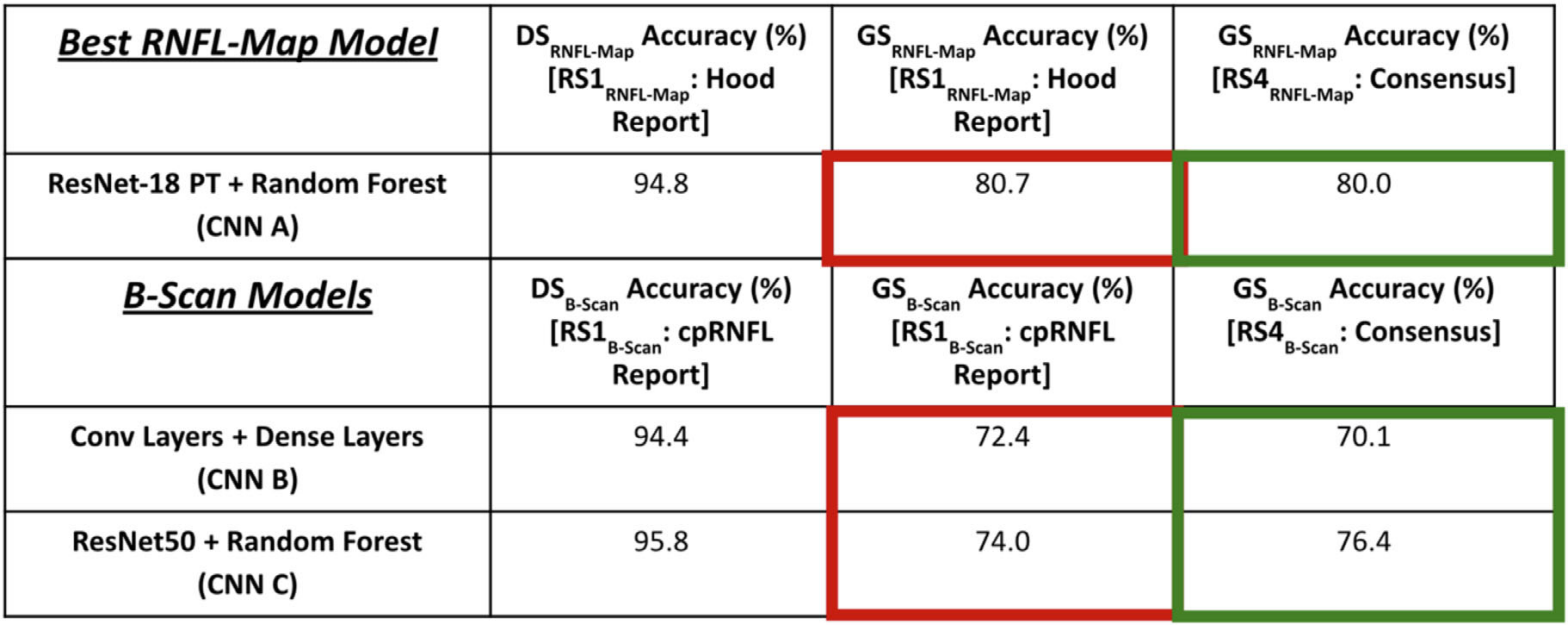

### Generalizability of RNFL Map and B-Scan Models Before Improvement

For both b-scan and RNFL map models, there was a significant decrease in performance when transferring to GS_RNFL-Map_/GS_B-Scan_ (Table 1, Columns 2 vs. 3). For the same RS (RS1_RNFL-Map_ and RS1_B-Scan_), the best-performing RNFL map model decreased from 94.8% to 80.7% (CNN A) (significant, *P* = 1.57 × 10^−4^, Wilcoxon signed rank test), whereas the b-scan models decreased from 94.4% to 72.4% (CNN B) (significant, *P* = 1.72 × 10^−4^, Wilcoxon signed rank test) and from 95.8% to 74.0% (CNN C) (significant, *P* = 1.33 × 10^−4^, Wilcoxon signed rank test). These are reductions of 14.1%, 22.0%, and 21.8%, respectively.

### Effect of Improvement Techniques on Generalizability

#### Impact of Data Augmentation and Multimodal Input

**Figure 5.**
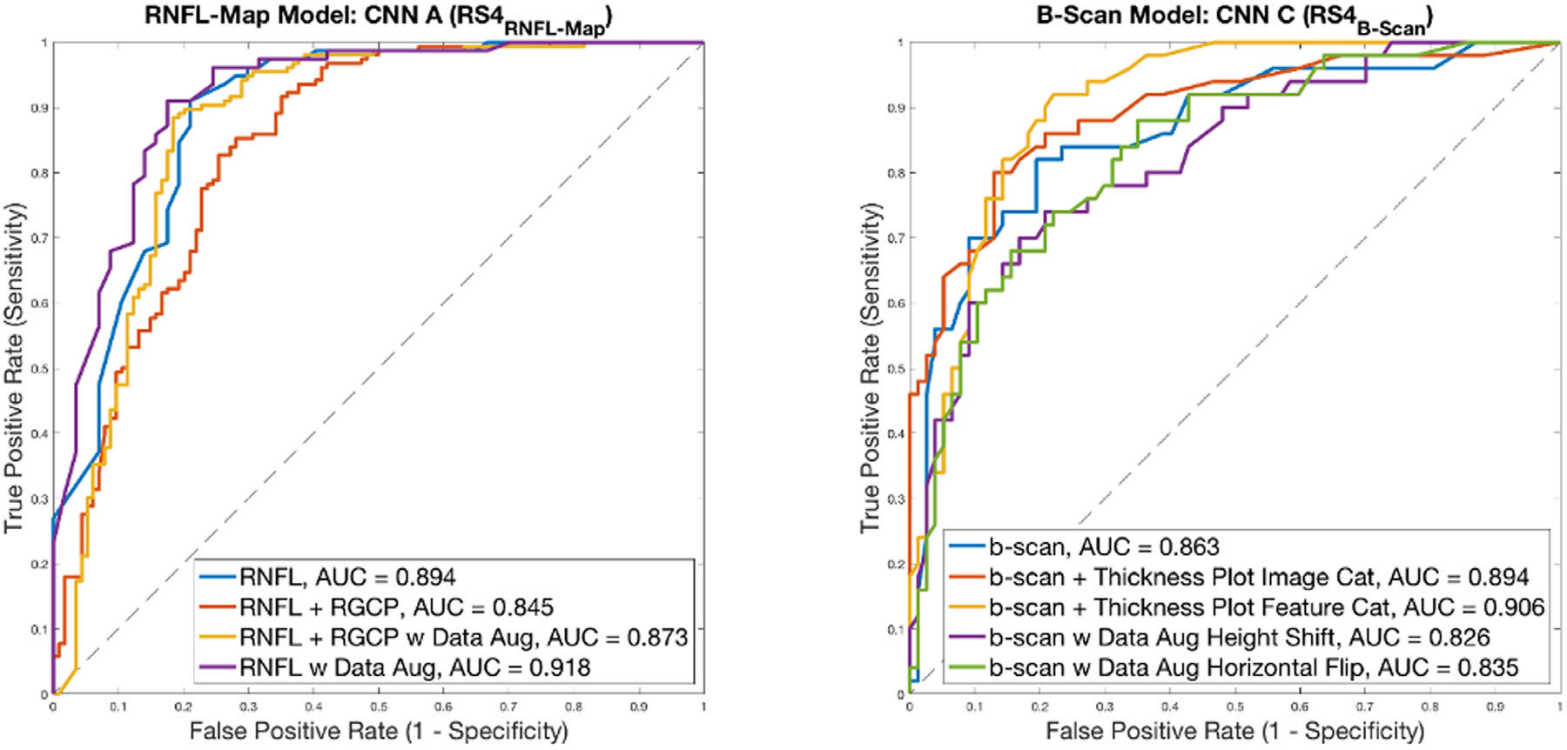
ROC curves showing impact of data augmentation and multimodal input for RNFL maps (left) and b-scans (right). AUC values are shown in legends for each curve.

Figure 5 contains receiver operating characteristic (ROC) curves showing the impact of multimodal input and data augmentation on generalizability for the RNFL map and b-scan models. Based on the area under the ROC curve (AUC) scores, RNFL probability map input alone with data augmentation resulted in the best generalizability for RNFL map models, with an AUC of 0.918 (95% confidence interval [CI], 0.866–0.970) (Fig. 5, left). This improvement can also be seen from Table 2 (compare columns 2 and 3). With data augmentation, CNN A accuracy increased by 5.2%, from 80.7% to 85.9% (significant, *P* = 3.60 × 10^−4^, Wilcoxon signed rank test). Multimodal input was less valuable in enhancing generalizability of the RNFL map model; AUC for RNFL + RGCP input was 0.845 (95% CI, 0.775–0.915) without data augmentation and was 0.873 (95% CI, 0.809–0.937) even with data augmentation.

**Table 2.** Impact of Data Augmentation on Generalizability Performance (GS_RNFL-Map_) for Best-Performing RNFL Map Model With RS1_RNFL-Map_ (red) and With RS4_RNFL-Map_ (Green)

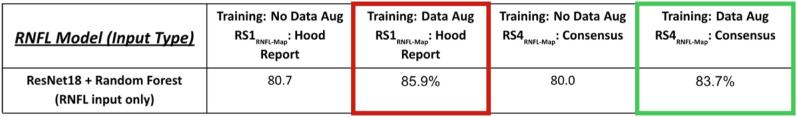

In contrast, data augmentation was less valuable in enhancing generalizability for the best b-scan model (resulting in lower AUCs of 0.826 [95% CI, 0.750–0.902] and 0.835 [95% CI, 0.762–0.909] for height shift and horizontal flip, respectively), but multimodal input (both feature concatenation and image concatenation approaches) resulted in the two highest AUC values of 0.906 (95% CI, 0.849–0.963) and 0.894 (95% CI, 0.834–0.954), respectively, for b-scan models (Fig. 5, right). Note that for these ROC curves, the RS used were RS4_RNFL-Map_ and RS4_B-Scan_, respectively.

#### Training on Confident Scans

A substantial, although not statistically significant, improvement in test accuracy performance on the generalizability set, GS_B-Scan_, was obtained by training the b-scan models, CNN B and CNN C, only on b-scans that the OCT expert rated as confident cases (i.e., the confident dataset, C-DS_B-Scan_), as can be seen in Table 3 (compare columns 2 and 3, CNN B). CNN B improved by 8.7%, from 72.4% to 81.1% (not significant, *P* = 0.477, Wilcoxon signed rank test).

**Table 3.** Impact of Confident Input on b-Scan Model Generalizability Performance (GS_B-Scan_) With RS1_B-Scan_ Shown in Red and With RS4_B-Scan_ Shown in Green.

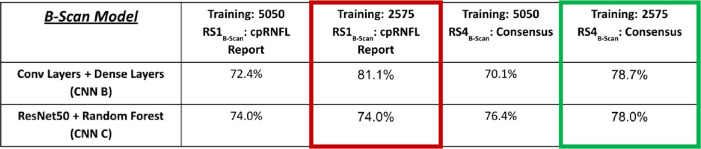

#### Impact of RS on Model Performance

**Figure 6.**
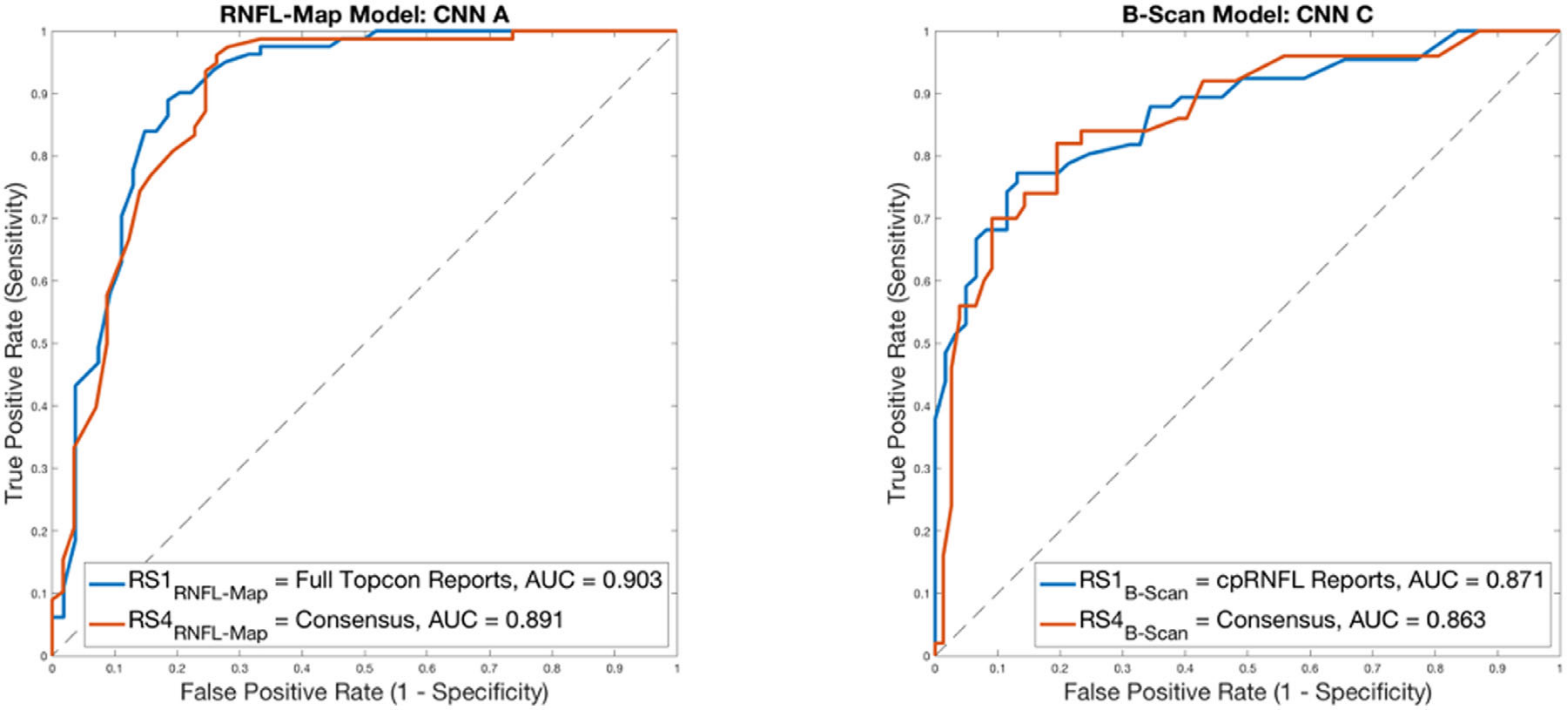
ROC curves showing impact of RS on model performance. For RNFL map input, the RS with higher AUC is full Hood reports, followed by consensus; for b-scan inputs, the RS resulting in higher AUC is cpRNFL reports, followed by consensus. However, there is no significant difference in AUC for both model types between RS1_RNFL-Map_/RS1_B-Scan_ and RS4_RNFL-Map_/RS4_B-Scan_, respectively (AUC values shown in legends).

Changing the RS had a significant impact on model accuracy for GS_RNFL-Map_ and GS_B-Scan_; however, it did not have a significant impact on model AUC. This can be seen especially for model accuracy on GS_RNFL-Map_ and GS_B-Scan_ using RS1_RNFL-Map_ and RS1_B-Scan_ vs. using RS4_RNFL-Map_ and RS4_B-Scan_, respectively. For RNFL map model CNN A, accuracy on GS_RNFL-Map_ was 80.7% with RS1_RNFL-Map_ and was 80.0% with RS4 _RNFL-Map_ (significant, *P* = 2.30 × 10^−5^, Wilcoxon signed rank test). For b-scan model CNN B, accuracy on GS_B-Scan_ was 72.4% with RS1_B-Scan_ and was 70.1% with RS4_B-Scan_ (not significant, *P* = 0.166, Wilcoxon signed rank test), and for CNN C, accuracy on GS_B-Scan_ was 74.0% with RS1_B-Scan_ and was 76.4% with RS4_B-Scan_ (significant, *P* = 0.002, Wilcoxon signed rank test). Figure 6 shows ROC curves for RNFL map input and for b-scan input with varying RS. For the RNFL map model, AUC was highest when RS was RS1_RNFL-Map_ (Hood report) at 0.903 (95% CI, 0.845–0.961), while RS4_RNFL-Map_ (consensus of experts) resulted in slightly lower AUC of 0.891 (95% CI, 0.831–0.951) (Fig. 6, left). This difference was not significant (*P* = 0.790, DeLong's test). The best-performing b-scan model (CNN C) parallels this, with the highest AUC of 0.871 (95% CI, 0.795–0.931) for RS1_B-Scan_ (cpRNFL reports) and slightly lower AUC of 0.863 (95% CI, 0.809–0.933) for RS4_B-Scan_ (consensus of experts), Figure 6 (right). This difference was not significant (*P* = 0.790, DeLong's test). Evident from Table 1 and these ROC curves is that CNN performance is highest when the RS used for acquiring ratings on model training data is the same as the RS used for acquiring ratings on model testing data (RS1_RNFL-Map_/RS1_B-Scan_ in our case), as shown by the red rectangles in Table 1. RS4_RNFL-Map_ and RS4_B-Scan_ result in minor reduction in performance for CNN A as well as for CNN B (green rectangles, Table 1), because the consensus RS was only used for acquiring ratings for model test data, but was not used during model training.

#### Combining Improvement Techniques and RS

The results for combining RS with optimal improvement techniques are shown in Table 2 for RNFL maps and in Table 3 for b-scans. Because the most effective improvement technique for RNFL probability maps was data augmentation, we show the glaucoma detection accuracy rates for RNFL map models with data augmentation using RS1_RNFL-Map_ (full Hood reports) and using RS4_RNFL-Map_ (consensus for RNFL map data) for RNFL input alone (Table 2). For RNFL map models, best performance is observed for the ResNet18 + random forest model with data augmentation and with RS1_RNFL-Map_, when the OCT expert viewed full Hood reports, the same RS used for RNFL map model training.

Because training on confident b-scans was most effective for b-scan models, the best parameters for b-scan inputs using RS1_B-Scan_ (cpRNFL reports) and RS4_B-Scan_ (consensus of experts for b-scan data) are shown in Table 3. Note highest CNN accuracy is achieved with CNN B with the C-DS training and RS1_B-Scan_, when the clinician only viewed the cpRNFL report for making a decision (same RS used for training).

## Discussion

One of the primary challenges of putting neural networks into practice is maintaining their generalizability to new test datasets. In particular, we need to know how well they will perform at new clinical sites when data are collected on different machines, by different operators, and for different patient populations. One should anticipate a decrease in model performance when transferring to an unseen dataset. In fact, we found that performance of our RNFL map and b-scan deep learning models was decreased by as much as 22.0% when evaluating the generalizability datasets without incorporation of any training optimizations.

**Figure 7.**
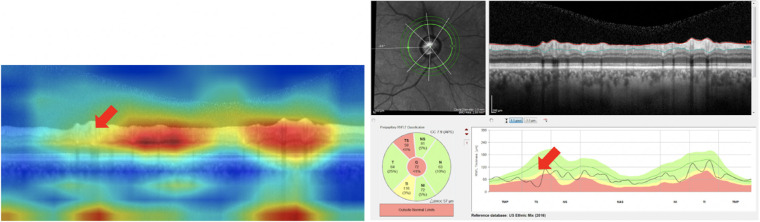
Attention Map^22^ visualization of b-scan (left) is an example of a False Negative (missed case); this attention map suggests that the CNN has missed a local defect (red arrow). Slight improvement in accuracy of multimodal b-scan images and thickness plots (similar in content to the cpRNFL report at right) may be due to the fact that thickness plots make local defect information more prominent (red arrow at right).

The primary purpose of this study was to assess and improve the generalizability of deep learning models trained to detect glaucoma. For both model types, we trained with multiple data augmentation techniques that had clinical face validity. Vertical shifting and horizontal flips were reasonable data augmentation choices for b-scans. For RNFL maps, because we know that the machine where the GS_RNFL-Map_ dataset was collected had an 8% to 10% scaling difference in image generation, we scaled images by 10% and also introduced horizontal flips (effectively changing right eyes into left eyes) and vertical flips (inverting the RNFL map),^19^ significantly improving model performance. For b-scan models, training on confident images (C-DS) improved model performance. This improvement can be attributed to the decrease in training noise afforded by only including scans that definitely belong to each class (glaucomatous vs. nonglaucomatous). Multimodal input played a role in slightly improving generalizability for b-scan images, possibly because the addition of the thickness plot increased the availability of information relating to local defects, which is important for glaucoma detection.^21^ This finding is supported by attention maps^22^ (which highlight image regions used by the CNN to make its decision) that suggest that local defects are missed in false-negative classifications using b-scan input alone, as shown in Figure 7.^17^ Also, vertically adjacent thickness plots and b-scans are similar to the commercial cpRNFL plots used by experts to detect glaucoma. Thus, the addition of thickness plot information may help by adding local defect information. In contrast, for RNFL map models, the best performance was observed for RNFL map input alone; single modality images in this case performed better than multiple modalities, possibly because features extracted from the combined inputs did not add new information to sufficiently separate glaucomatous from not glaucomatous images, but instead added noise to the classification decision. (See Discussion of false positives and false negatives using an RNFL map visualization technique, as well as Supplementary Figure S3 and Supplementary Table S4). This finding suggests that more knowledge of OCT report subimages used by experts to bias CNN feature extraction toward those regions may enhance performance for RNFL map models.^19^

Our second purpose here was to explore the consequences of using different RS. Interestingly, there was a significant difference in accuracy between RS1_RNFL-Map_/RS1_B-Scan_ (full Hood reports/cpRNFL reports) and RS4_RNFL-Map_/RS4_B-Scan_ (consensus for RNFL maps/consensus for b-scans) for two of the three models (CNN A and CNN C), whereas model AUCs were comparable for these two models. The intergrader kappa statistic between RS1_RNFL-Map_ and RS4_RNFL-Map_ was consistent with model AUCs, at 0.954, indicating near perfect agreement; similarly, the intergrader kappa statistic between RS1_B-Scan_ and RS4_B-Scan_ was 0.702, indicating fair agreement. Even though intergrader agreement is relatively high between RS1 and RS4 in both cases, the additional variance between graders for the consensus ratings (both RS4 cases) may have contributed to the significant difference in accuracy between models trained on these two RS. We did expect RS1_RNFL-Map_/RS1_B-Scan_ to exhibit higher accuracies, because this RS was used both for training and testing. However, at the same time (based on comparable AUCs with differing RS shown in Fig. 6), it seems that our models are well-buffered and robust to changes in RS. This divergence in significance between accuracy and AUC highlights the important role played by the RS. In particular, this study suggests the need for training and testing with a similarly defined RS, as model performances were significantly better for two of the three models presented here for datasets based on RS1_RNFL-Map_/RS1_B-Scan_ (and models were trained with RS1_RNFL-Map_/RS1_B-Scan_). Any information available to graders while establishing ground truth for the training dataset should be available to graders when assigning labels to the test dataset as well. These findings suggest that the RS should be one that is consistent throughout training and testing as well as clinically optimal (i.e., built on as much information as possible) to ensure generalizable CNN performance and the greatest clinical accuracy for patient diagnoses. In clinical terms, future commercially available CNNs should be developed using RS that in fact replicate the wealth of information clinicians use in practice, in particular the full OCT data. We acknowledge that tailoring RS used for training and testing may only be feasible when CNNs are being developed at the same facility where patient data are being collected; in cases when CNNs are developed elsewhere, training details such as RS, training dataset size, and optimizations used should be questioned and clarified before use to gauge expected generalizability to new data.

## Future Directions

Combining optimal strategies for each model type may further enhance performance; for example, using data augmentation specifically on confidently trained b-scan models or training RNFL models with data augmentation only on confidently rated RNFL maps are potential combinations for future exploration. To further take advantage of multimodal input, CNN ensemble approaches^19^ (averaging the predictions of multiple models, each taking as input a separate image type)—as opposed to the multimodal feature extraction/concatenation approaches described in this article—may also enhance generalizable performance. In addition, accuracy may be improved by combining multimodal structure and function information by extracting features from visual fields and OCT images, instead of RNFL and RGCP maps or b-scans and thickness plots as was done here. Finally, using clinically informative regions of interest, such as temporal regions of the RNFL in b-scans, may enhance b-scan model performance.

## Conclusions

The generalization accuracy of CNNs can be improved with data augmentation, multiple input image modalities, and training on images with confident ratings. Specifically, for RNFL map models, incorporating data augmentation during training improved generalizability performance, and RNFL map input alone achieved better performance than combined RNFL and RGCP maps. For b-scan models, training on confident scans and multimodal approaches improved generalization accuracy to different extents. CNNs trained and tested with the same RS achieved best accuracy, suggesting that choosing a thorough and consistent RS for training and testing improves generalization to new datasets. Strategies for enhancing the generalizability of CNNs and for choosing optimal RS should be standard practice for CNNs before their deployment for glaucoma detection.

## Supplementary Material

Supplement 1
