# Supplementary material for: Strategies to Improve Convolutional Neural Network Generalizability and Reference Standards for Glaucoma Detection From OCT Scans
Source: Transl Vis Sci Technol. 2021 Apr 15;10(4):16. doi: 10.1167/tvst.10.4.16 (PMC8054628; doi:10.1167/tvst.10.4.16)
Supplement: Supplement 1 [file tvst-10-4-16_s001.pdf]

## SUPPLEMENTARY INFORMATION

## Methods:

### *Table S1a: Dataset Composition for RNFL-Map Model ($RS4_{RNFL-Map}$ for $GS_{RNFL-Map}$)*

| NG vs. G | RNFL Map Data Distribution | | | |
|---|---|---|---|---|
| | $DS_{RNFL-Map}$ training | $DS_{RNFL-Map}$ validation | $DS_{RNFL-Map}$ testing | $GS_{RNFL-Map}$ testing |
| **NG** | 285 | 118 | 141 | 78 |
| **G** | 110 | 27 | 56 | 57 |
| **Totals** | 395 | 145 | 197 | 135 |

### *Table S1b: Dataset Composition for B-Scan Models ($RS4_{B-Scan}$ for $GS_{B-Scan}$)*

| | B-Scan Data Distribution (5050) | | | | B-Scan Data Distribution (2575) | | | |
|---|---|---|---|---|---|---|---|---|
| | $DS_{B-Scan}$ training | $DS_{B-Scan}$ validation | $DS_{B-Scan}$ testing | $GS_{B-Scan}$ testing | C-$DS_{B-Scan}$ training | C-$DS_{B-Scan}$ validation | C-$DS_{B-Scan}$ testing | $GS_{B-Scan}$ testing |
| **NG** | 297 | 95 | 82 | 77 | 265 | 90 | 89 | 77 |
| **G** | 178 | 59 | 60 | 50 | 154 | 52 | 53 | 50 |
| **Totals** | 475 | 154 | 142 | 127 | 419 | 142 | 142 | 127 |

### *Figure S1: B-Scan CNN Architectures Schematic*

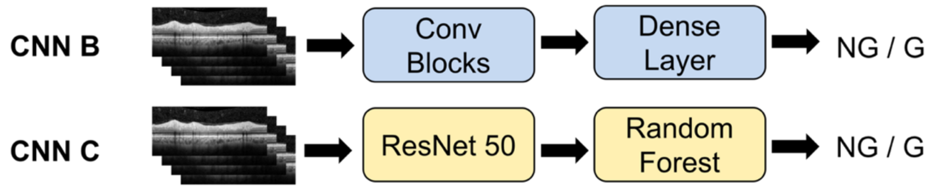


### *Figure S2: Architecture Details of OCT-Trained B-Scan Model, CNN B*

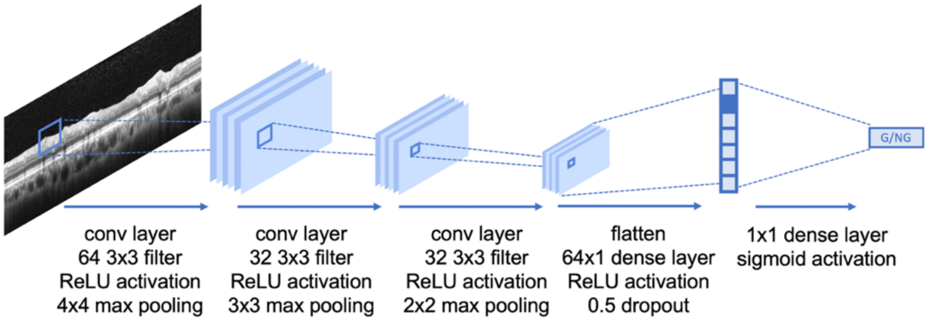

We provide here the relative strengths of CNN B:

(1) **Good for custom dataset:** CNN B can be trained from scratch using a custom dataset. Medical images are different from natural images of ImageNet.  Suppose a lab has a relatively small image training dataset, and the image modality is different from natural images in ImageNet; it is worth considering CNN B as a feasible approach to compare model performance with CNN C.

(2) **Efficiency:** CNN B can be trained in a short time. It is an efficient approach due to its simple architecture.

We provide here the relative weaknesses of CNN B:

(1) **Hyperparameter tuning:** effective performance of CNN B requires tuning parameters (regularization/dropout, kernel filter size, activation functions, etc.) based on validation results.

(2) **Underfitting:** There might be an underfitting problem due to the limited number of parameters. In contrast, CNN C may overfit the training set.

**Results:**

***Table S2: Results of Training and Testing on Confident B-Scans***

| RS | **RS1$_{B\text{-}Scan}$: cpRNFL Report** | | | | | | **RS2$_{B\text{-}Scan}$: Heidelberg Report** | | | | | | **RS3$_{B\text{-}Scan}$: B-Scan** | | | | | |
|---|---|---|---|---|---|---|---|---|---|---|---|---|---|---|---|---|---|---|
| Scan Rating | NG < 50, G > 50 | | | NG < 25, G > 75 | | | NG < 50, G > 50 | | | NG < 25, G > 75 | | | NG < 50, G > 50 | | | NG < 25, G > 75 | | |
| Metrics/Models | ACC | FP | FN | ACC | FP | FN | ACC | FP | FN | ACC | FP | FN | ACC | FP | FN | ACC | FP | FN |
| **CNN B 5050** | 72.44% | 0 | 35 | **76.42%** | 0 | 25 | 64.57% | 5 | 40 | 73.96% | 0 | 25 | 66.14% | 1 | 42 | 69.88% | 1 | 24 |
| **CNN C 5050** | 74.02% | 1 | 32 | **79.25%** | 0 | 22 | 73.23% | 2 | 32 | 77.08% | 0 | 22 | 66.93% | 2 | 40 | 75.90% | 0 | 20 |
| **CNN B 2575** | 81.10% | 1 | 23 | **85.85%** | 1 | 14 | 73.23% | 6 | 28 | 83.33% | 1 | 15 | 77.95% | 0 | 28 | 83.13% | 0 | 14 |
| **CNN C 2575** | 74.02% | 1 | 32 | **78.30%** | 1 | 22 | 70.87% | 3 | 34 | 77.08% | 1 | 21 | 69.29% | 1 | 38 | 75.90% | 0 | 20 |

***Table S3: DS$_{RNFL\text{-}Map}$/DS$_{B\text{-}Scan}$ and GS$_{RNFL\text{-}Map}$/GS$_{B\text{-}Scan}$ Accuracies for all RS***

| ***Best RNFL-Map Model*** | **$DS_{RNFL\text{-}Map}$ Accuracy (%) [$RS1_{RNFL\text{-}Map}$: Hood Report]** | **$GS_{RNFL\text{-}Map}$ Accuracy (%) [$RS1_{RNFL\text{-}Map}$: Hood Report]** | **$GS_{RNFL\text{-}Map}$ Accuracy (%) [$RS2_{RNFL\text{-}Map}$: RNFL + RGCP probability maps]** | **$GS_{RNFL\text{-}Map}$ Accuracy (%) [$RS3_{RNFL\text{-}Map}$: RNFL probability map only]** | **$GS_{RNFL\text{-}Map}$ Accuracy (%) [$RS4_{RNFL\text{-}Map}$: Consensus]** |
|---|---|---|---|---|---|
| **ResNet-18 PT + Random Forest (CNN A)** | 94.8 | 80.7 | 77.8 | 83.0 | 80.0 |
| ***B-Scan Models*** | **$DS_{B\text{-}Scan}$ Accuracy (%) [$RS1_{B\text{-}Scan}$: cpRNFL Report]** | **$GS_{B\text{-}Scan}$ Accuracy (%) [RS1B: cpRNFL Report]** | **$GS_{B\text{-}Scan}$ Accuracy (%) [$RS2_{B\text{-}Scan}$: HE Full Report]** | **$GS_{B\text{-}Scan}$ Accuracy (%) [$RS3_{B\text{-}Scan}$: B-scan only]** | **$GS_{B\text{-}Scan}$ Accuracy (%) [$RS4_{B\text{-}Scan}$: Consensus]** |
| **Conv Layers + Dense Layers (CNN B)** | 94.4 | 72.4 | 64.6 | 66.1 | 70.1 |
| **ResNet50 + Random Forest (CNN C)** | 95.8 | 74.0 | 73.2 | 66.9 | 76.4 |

## Discussion:

### *Visualization Technique Helps to Explain False Positives and False Negatives*

Visualizing Features Used by CNNs to Detect Glaucoma in RNFL Maps to Speculate Reasons for False Positives and False Negatives: For RNFL maps, we used a visualization approach called Grad-CAMs (Gradient Weighted Class Activation Maps),[1] which highlight regions in images that contribute positively to a neural network's classification decision. To make Grad-CAMs more quantitative, we compared Grad-CAMs to OCT images superimposed by visual field points showing regions of structure-function agreement,[2,3] we found that regions highlighted in Grad-CAMs are also regions with abnormal structure and abnormal function agreement (aS-aF). Figure S3 below shows an example of both a Grad-CAM for an RNFL probability map (top left) and an aS-aF diagram (top right) with RNFL probability map, superimposed visual field (VF) points, and aS-aF locations marked by VF points circumscribed by diamonds (24-2 VF locations) or squares (10-2 VF locations).

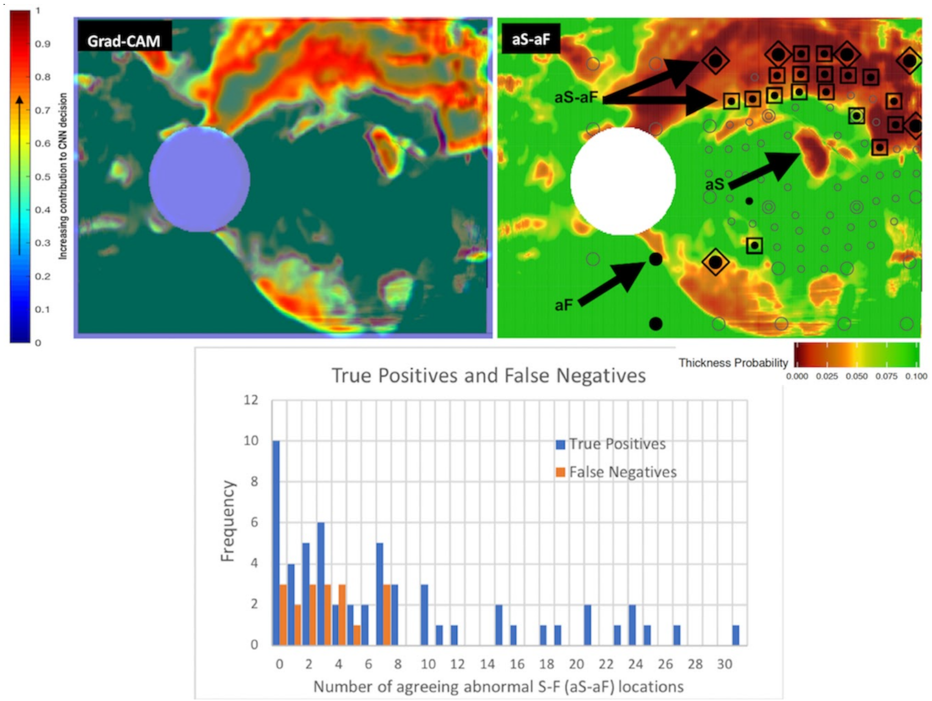


**Figure S3:** Above left panel shows a Grad-CAM (regions contributing to CNN classification of glaucoma highlighted in red and yellow); the corresponding RNFL map is shown in the upper right: OCT structural information is superimposed by VF functional information, and regions of abnormal structure and abnormal function agreement (aS-aF) are shown by VF points circumscribed by squares and diamonds. Bottom panel contains a histogram showing that the overwhelming number of aS-aF locations are in True Positive RNFL maps (detected as glaucomatous by AI and labeled as glaucomatous by the clinician), like this one.[4]

We found that the number of aS-aF locations is significantly greater ($p < 0.05$, Mann-Whitney Test) for *True Positives* (correctly identified glaucoma cases) than for *False Negatives* (missed glaucoma cases) across all RNFL maps in $GS_{RNFL\text{-}Map}$ studied here.[4]

Below is a table of false positives and false negatives that are currently arrived at by our RNFL-map and b-scan models; they have a tendency currently to result in many more false negatives (misses) than false positives. This can be explained by the Grad-CAM and structure-function analysis described above; many of these misses are subtle/edge cases, where damage is not extreme, so the models are missing regions where there is no significant structure-function agreement. Therefore, the models may improve with the incorporation of more such ambiguous examples in training or by designing specific

filters to enable our CNNs to recognize patterns of features characteristic of these subtle cases.

### Table S4: False Positives and False Negatives for RNFL-Map and B-Scan Models

| **Model (Below)** | **False Positives (FPs/Total NG) Training: 2575 $\text{RS4}_{\text{B-Scan}}$: Consensus** | **False Negatives (FNs/Total G) Training: 2575 $\text{RS4}_{\text{B-Scan}}$: Consensus** |
|---|---|---|
| **Conv Layers + Dense Layers (B-Scan CNN B)** | 8 (11.1%) | 19 (34.5%) |
| **ResNet50 + Random Forest (B-Scan CNN C)** | 4 (5.6%) | 24 (43.6%) |
| **Best RNFL Model Below** | **False Positives (FPs/Total NG) RNFL input w/ Data Aug $\text{RS4}_{\text{RNFL-Map}}$: Consensus** | **False Negatives (FNs/Total G) RNFL input w/ Data Aug $\text{RS4}_{\text{RNFL-Map}}$: Consensus** |
| **ResNet18 + Random Forest (CNN A)** | 2 (2.6%) | 20 (35.1%) |

### Table S5: Most Commonly-used Abbreviations for Experiments Conducted

| OCT Image Type | Training Dataset (DS) | Generalizability Set (GS) | RS1 (Reference Standard based on single expert viewing Topcon reports for RNFL maps or Heidelberg cpRNFL reports for b-scans) | RS4 (Reference Standard based on consensus of multiple experts viewing OCT and VF information) |
|---|---|---|---|---|
| **RNFL Maps** | $\text{DS}_{\text{RNFL-Map}}$ | $\text{GS}_{\text{RNFL-Map}}$ | $\text{RS1}_{\text{RNFL-Map}}$ | $\text{RS4}_{\text{RNFL-Map}}$ |
| **B-Scans** | $\text{DS}_{\text{B-Scan}}$ | $\text{GS}_{\text{B-Scan}}$ | $\text{RS1}_{\text{B-Scan}}$ | $\text{RS4}_{\text{B-Scan}}$ |

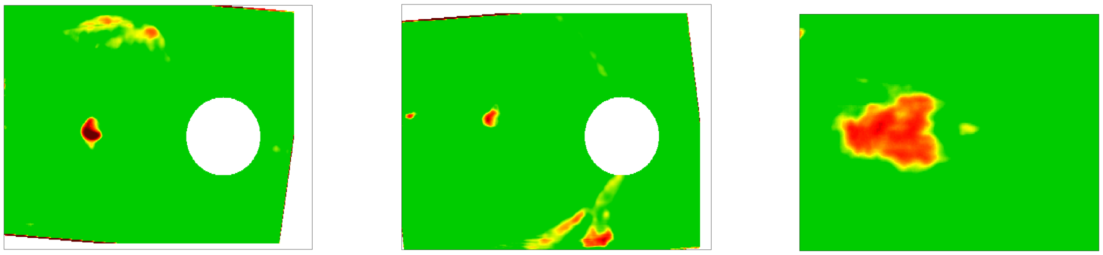

**Figure S4:** Images disagreed upon by $\text{RS1}_{\text{RNFL-Map}}$ and $\text{RS4}_{\text{RNFL-Map}}$. There are in fact only 3 RNFL maps for which the ratings are reversed between $\text{RS1}_{\text{RNFL-Map}}$ and $\text{RS4}_{\text{RNFL-Map}}$; the left-most two are indeed subtle/mild cases, and the right-most one contains a pattern seen in both healthy controls and patients. These 3 images were all categorized as nonglaucomatous by $\text{RS1}_{\text{RNFL-Map}}$ and were categorized as glaucomatous by $\text{RS4}_{\text{RNFL-Map}}$.

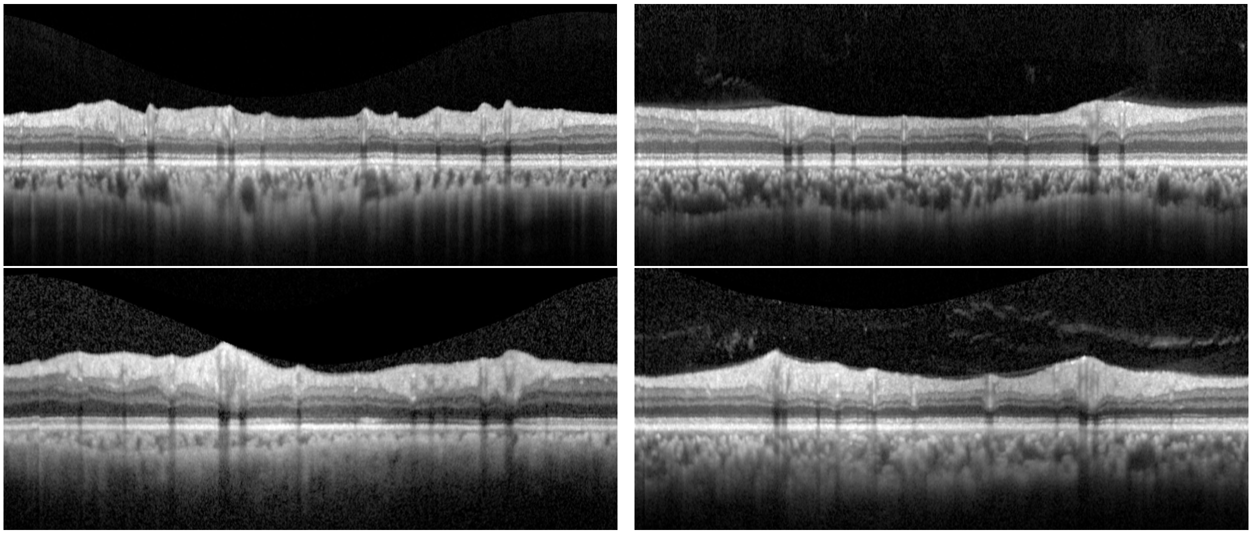

**Figure S5:** (Top) Two examples that $RS1_{B\text{-}Scan}$ labeled glaucomatous while $RS4_{B\text{-}Scan}$ labeled non-glaucomatous; (bottom) two examples that $RS1_{B\text{-}Scan}$ labeled non-glaucomatous while $RS4_{B\text{-}Scan}$ labeled glaucomatous. The b-scans that $RS1_{B\text{-}Scan}$ and $RS4_{B\text{-}Scan}$ disagreed on are also suspects or mild cases. In the top row, the left example has some local defects, and the right example has a thinning retinal nerve fiber layer; these are typical glaucomatous features. In the bottom row, the glaucomatous defects are not very obvious in these b-scans. The extra information used by experts in $RS4_{B\text{-}Scan}$ may have helped to clarify these boundary cases.

**References:**


1. Selvaraju, R, Cogswell, M, Das A, Vedantam R, Vedantam, Parikh D. Batra D. Grad-CAM: Visual explanations from deep networks via gradient-based localization." *International Conference on Computer Vision*, 2017.
2. Tsamis E, Bommakanti NK, Sun A, Thakoor KA, De Moraes CG, Hood DC. An Automated Method for Assessing Topographical Structure–Function Agreement in Abnormal Glaucomatous Regions." *Translational Vision Science and Technology*, 2020;9(4),14-14.
3. Hood DC, Tsamis E, Bommakanti NK, Joiner DB, Al-Aswad LA, Blumberg DM, Cioffi GA, Liebmann JM, and De Moraes. Structure-Function Agreement Is Better Than Commonly Thought in Eyes With Early Glaucoma. *Investigative ophthalmology & visual science,* 60, no. 13 (2019): 4241-4248.
4. Thakoor, KA, Tsamis, EM, De Moraes, CG, Sajda, P, Hood, DC, 2020. Impact of Reference Standard, Data Augmentation, and OCT Input on Glaucoma Detection Accuracy by CNNs on a New Test Set. *Investigative Ophthalmology & Visual Science*. 2020;61(7):4540-4540.
